# Seizure following the Use of the COX-2 Inhibitor Etoricoxib

**DOI:** 10.1155/2017/1410759

**Published:** 2017-01-22

**Authors:** Valentina Arnao, Marianna Riolo, Brigida Fierro, Paolo Aridon

**Affiliations:** Dipartimento di Biomedicina Sperimentale e Neuroscienze Cliniche, Università degli Studi di Palermo, Palermo, Italy

## Abstract

We describe a case of epileptic seizures occurring after the use of a COX-2 inhibitor. A 61-year-old man was admitted to our department because of a generalized tonic-clonic seizure. EEG showed generalized slowdown of the activity. Neuroimaging and blood samples studies did not evidence alterations, but a careful pharmacological history revealed that the patient had taken the COX-2 inhibitor etoricoxib to treat lumbago few days before the onset of clinical symptoms. No seizures were reported after etoricoxib discontinuation and an EEG resulted to be normal two months after this.* Conclusion*. Knowing the pharmacological history of a patient is important for understanding the clinical presentation and selecting appropriate treatment. This is, to the best of our knowledge, the first reported case of generalized seizures associated with the use of COX-2 inhibitors.

## 1. Introduction

Epileptic seizures can be the consequence of different causes, including brain lesions, electrolyte or metabolic abnormalities, and adverse drug events. There are diverse pharmacological molecules that can induce seizures, including tricyclics, venlafaxine, bupropion, and isoniazid. To our knowledge, COX-2 inhibitors have never been included on this list.

## 2. Case Report

A 61-year-old man arrived to our emergency room suffering from generalized tonic-clonic seizure followed by confusion upon awakening. The patient had a history of parossistic atrial fibrillation and two syncopal episodes due to sick sinus syndrome. Being so, a pacemaker was implanted and the patient initiated anticoagulant therapy. In 2009, a fall provoked a bilateral subdural hematoma which completely disappeared within six months. At the emergency room, a cardiological evaluation confirmed that the pacemaker was working properly and it excluded a cardiogenic syncope. Moreover, the patient denied sleep deprivation, alcohol abuse, and illicit drug use. A more detailed pharmacological history revealed that, during the three days prior to the generalized tonic-clonic episode, he had taken a COX-2 inhibitor (etoricoxib 90 mg/day) for the first time ever to treat lumbago. Neurological examination revealed a slight right faciobrachial hemiparesis and a postural tremor of the right arm, but, after a couple of hours, the neurological examination resulted to be normal. A brain CT scan showed an old ischemic lesion in the left semioval region. Electroencephalogram (EEG) indicated the presence of generalized and nonspecific slowing of electrical activity. Serum examination did not reveal any electrolyte alterations or other metabolic abnormalities. MRI could not be performed because of the presence of the pacemaker, but a brain CT scan performed on day three did not reveal any new lesions. Subsequently, levetiracetam 500 mg twice a day was started. At one-month follow-up, the patient was not taking COX-2 inhibitors anymore, he did not report any seizures, and an EEG showed normal brain activity. For this, levetiracetam was gradually reduced and eventually stopped. At six-month follow-up, no seizures were described and an EEG, the first after the antiepileptic drug withdrawal, produced normal results. One year later, the patient was in good health and continued to report being without seizures.

## 3. Discussion

Selective COX-2 inhibitors (celecoxib, rofecoxib, valdecoxib, and etoricoxib) are approved for use in rheumatoid arthritis, osteoarthritis, and acute pain. These drugs demonstrate at least a 200- to 300-fold selectivity for inhibition of COX-2 over COX-1; this leads to a more powerful effect on decreasing inflammation and a minimum toxicity (particularly gastroduodenal erosion), because there is no effect on the constitutive COX-1 isoform. From a chemical point of view, selective COX-2 inhibitors possess 1,2-diarylsubstitution on a central heterocyclic or carbocyclic ring system with a characteristic methanesulfonyl, sulfonamido, azido, methanesulfonamide, or pharmacophore-based tetrazole group on one of the aryl rings that play a crucial role on COX-2 selectivity. Etoricoxib, in particular, belongs to the 6-membered core group and, as other 1,2-diarylpyridine derivatives, it has shown good COX-2 inhibitor potency and selectivity.

The principal benefit associated with selective COX-2 inhibitors is a production of comparable analgesia and anti-inflammatory effects to the nonselective NSAIDs, with fewer symptomatic gastric and duodenal ulcers and a decrease in gastrointestinal symptoms. Limited data are available concerning toxicity associated with COX-2 inhibitors outside the gastrointestinal tract [1]. Some studies have suggested that the COX-2 enzyme has a significant role in renal development and function, so the use of its inhibitor can cause an acute renal failure. In fact, there is evidence that selective COX-2 inhibitors adversely affect renal function in patients at risk [2]. They can also cause anaphylactoid reaction, cardiovascular effects, and aseptic meningitis [3]. Regarding etoricoxib, postmarketing cases of induced immune haemolytic anemia [4], toxic epidermal necrolysis, and an erythema-multiform-like eruption have been reported [5].

In our case, in light of medical history and the concomitant use of anticoagulation therapy, the first hypothesis to exclude was hemorrhagic event. As brain CT ruled out both hemorrhagic and ischemic diseases, the clinical presentation suggested the occurrence of a first seizure in an adult. In fact, the focal symptoms and the global slowdown of brain electrical activity were secondary to the epileptic event (as a matter of fact, both improved soon after). No association of seizure to the patient's lumbago was found. Limb-shaking TIAs are unusual, especially upon awakening, and they generally occur in patients with severe carotid occlusive disease. This was not the case for our patient. Moreover, considering that the main blood parameters resulted to be normal and a malfunction of the pacemaker was excluded, the clinical manifestation could not have been a nonepileptic event. As the risk of a recurrent seizure is greater within the first two years after a first seizure for adults presenting with an unprovoked first seizure (21–45%) [6], an epileptic drug was started with the warning not to take COX-2 inhibitors.

As far as we know, etoricoxib-induced seizures have not been reported in literature; but, between January 2004 and October 2012, three individuals taking etoricoxib were reported to have epileptic seizures to the Food and Drug Administration (FDA).

Few experimental studies on rats have shown an antiepileptic role of COX-2 inhibitors in reducing seizure-induced neuroinflammation and neuronal hyperexcitability [7]. Many medications are known to be associated with an increased risk of seizures [8], at both therapeutic and toxic concentrations [9], but the underlying mechanisms are poorly understood. In fact, drug-induced seizures can occur as a direct result of altering neural pathways and specific excitatory or inhibitory transmitters and receptors within those pathways; nevertheless, because of complex interactions, no single mechanism exists for explaining all cases of drug-induced seizures. Studies have estimated that 6% of new-onset seizures and up to 9% of status epilepticus cases are due to drug toxicity [10]. Phenothiazine, tricyclic antidepressants, salicylate overdoses, chemotherapeutic agents, baclofen, and an abrupt withdraw from benzodiazepines or barbiturates have been clearly linked to seizures [8]. However, a link between etoricoxib and seizure has never been described, maybe due to the fact that a biological relation is difficult to determine.

Despite the fact that the role of inflammatory mediators in the pathogenesis of epilepsy is well known, the role of prostaglandin in epilepsy continues to be debated. Prostaglandins, which markedly increase following seizures, may contribute to epileptogenesis and a reduction of the seizure threshold [11].

The functions of prostaglandins in epileptogenesis have been studied; however, the role of COX-2 in epilepsy remains unclear. Conflicting results have been obtained in laboratory settings when treatment with a COX-2 inhibitor was administered before or after induced seizures. It seems that COX-2 may play an early neuroprotective and late neurotoxic role following seizures [12]. This bidirectional role could also be explained by the diversity of prostaglandins (e.g., PGD2 exhibits anticonvulsive functions but PGE2 causes epileptogenesis). In fact, PGE2 synthase activity is tightly coupled with COX-2. In our case, we can hypothesize that etoricoxib may have induced an unbalanced production of prostaglandins (e.g., PGE2) which in turn may have led to neuronal hyperexcitability by increasing ion channel permeability or enhancing glutamate release from astrocytes.

In order to document a causal association between etoricoxib use and seizure, dependency between the two must be proven. This can be done by considering any temporal and dose-response relationship, any replication of findings, biological plausibility, the exclusion of alternative explanations, a reduction of the events after the elimination of exposure to the factor, specificity of the association, and consistency with current knowledge [13]. In our case, there was an observed temporal relationship, lack of events after cessation of exposure to the drug, and an exclusion of alternative explanations. Ethical and clinical considerations did not allow us to reintroduce etoricoxib to investigate for recurrence.
